# *Salmo kottelati*, a new species of trout from Alakır Stream, draining to the Mediterranean in southern Anatolia, Turkey (Teleostei, Salmonidae)

**DOI:** 10.3897/zookeys.462.8177

**Published:** 2014-12-10

**Authors:** Davut Turan, Esra Doğan, Cüneyt Kaya, Mahir Kanyılmaz

**Affiliations:** 1 Recep Tayyip Erdogan University, Faculty of Fisheries and Aquatic Sciences, 53100 Rize, Turkey; 2 Mediterranean Fisheries Research Production and Training Institute, 07000, Antalya, Turkey

**Keywords:** Anatolia, fish distribution, taxonomy, *Salmo*, new species

## Abstract

*Salmo kottelati* **sp. n.**, is described from Alakır Stream (Mediterranean basin) in Turkey. It is distinguished from other Anatolian *Salmo* species by a combination of the following characters (none unique to the species): general body colour greenish to silvery in life; 7–9 parr marks along lateral line; four dark bands on flank absent in both sexes; black ocellated spots few, present only on upper part of flank in individuals smaller than 160 mm SL but in larger both males and females black spots numerous and located on back and middle and upper part of flank; red spots few to numerous, scattered on median, and half of lower and upper part of flank; head long (length 29–33% SL in males, 26–32 in females); mouth large (length of mouth gape 13–19% SL in males, 12–15 in females); maxilla long (length 10–13% SL in males, 8–12 in females); 105–113 lateral line scales; 24–29 scale rows between lateral line and dorsal-fin origin, 17–19 scale rows between lateral line and anal-fin origin; 13–15 scales between lateral line and adipose-fin insertion.

## Introduction

*Salmo trutta* Linnaeus, 1758 has long been considered to be a polymorphic species widely distributed throughout Europe and the Middle East reaching in south and south-east to the Atlas Range (Morocco, Algeria) and to the upper Amu-Darya drainage in Afghanistan (Kottelat 1997). Since many years, a number of subspecies of *Salmo trutta* or distinct species then assigned to *Salmo trutta* have been described. Most authors considered *Salmo trutta* to be a very variable species forming three major ecotypes (sea migratory, lacustrine, and riverine or brook).

Some forms or subspecies of *Salmo trutta* distributed in Europe and Asia were resurrected to the species level by Kottelat (1997). Later, species status of some North African species was discussed by Delling and Doadrio (2005) and of Balkan ones by Delling (2003, 2011). Kottelat and Freyhof (2007) tentatively recognised 29 species from European waters and mentioned that the status of several populations and nominal species was still not clear.

Tortonese (1955) reviewed the trouts of Anatolia and reported four subspecies of *Salmo trutta*: *Salmo trutta labrax* Pallas, 1814 from the Çoruh River (Black Sea basin), Lake Çıldır in the Kura drainage (Caspian Sea basin), and Uludağ mountains (Marmara Sea basin); *Salmo trutta caspius* Kessler, 1877 from the Kura River (Caspian Sea basin); *Salmo trutta macrostigma* (Duméril, 1858) from the Çoruh River (Black Sea basin), and the Çatak Stream (the Tigris system) and described *Salmo trutta abanticus* Tortonese, 1955, as a new subspecies, from Lake Abant (a closed lake in northwest Anatolia). Distribution area of *Salmo trutta macrostigma* was later considered as including, besides the Çoruh River, the Aegean Sea basin, the Marmara Sea basin, the Trace region, the Mediterranean basin, and the Tigris-Euphrates drainage (Geldiay and Kähsbauer 1967; Kelle 1978; Kuru 1975, 2004; Balık 1984; Bardakçı et al. 1994; Küçük 1997). *Salmo trutta labrax* was commonly reported from the streams and rivers flowing to the southeastern shore of the Black Sea, and *Salmo trutta caspius* from the Kura drainage (Aras 1974; Kuru 1975, 2004; Kutrup 1994; Geldiay and Balık 2007, Tabak et al. 2002; Turan 2003). In addition, *Salmo platycephalus* Behnke, 1968 was described from the upper Seyhan drainage (Behnke 1968; Kuru 2004; Schöffmann 2004).

**Figure 1.**
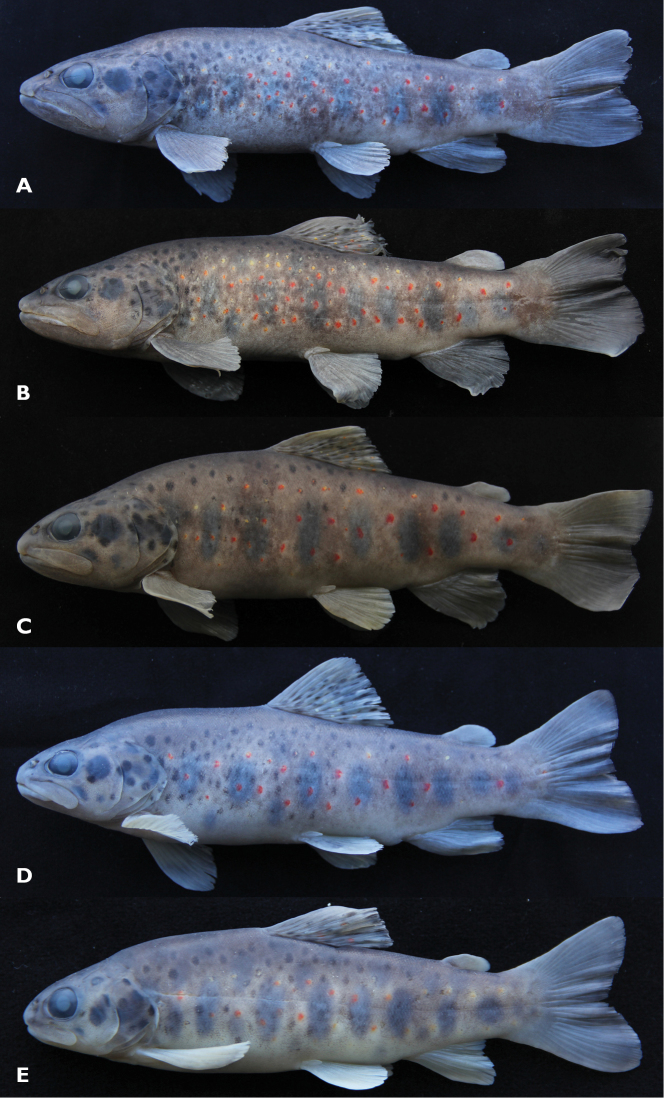
*Salmo kottelati*; Turkey: Antalya Province: Alakır stream **a** FFR 03180, holotype, 205 mm SL, male **b** FFR 03181, paratype, 207 mm SL, male **c** FFR 03181, paratype, 208 mm SL, female **d** FFR 03181, paratype, 175 mm SL, female **e** FFR 03181, paratype, 98 mm SL, Juvenile.

Turan et. al (2010) surveyed all rivers and streams draining to the Black Sea coast in Anatolia, and recognised three morphologically distinct groups of *Salmo* populations. These groups were identified by earlier authors as *Salmo trutta labrax* (drainages of south-eastern Black Sea coast), *Salmo trutta macrostigma* (Çoruh River drainage), and *Salmo trutta abanticus* (the outlet of the Abant Lake in the Bolu Province). Turan et al. (2010) compared the samples from the Black Sea coast of Anatolia with a sample of true *Salmo labrax* from the Ulu-Uzen River in Crimea (geographically close to one of the sites of the type locality of *Salmo labrax*, Biyuk-ozen River in Kacha River upper reaches in Crimea) and a sample from the Khosta River in Krasnodar Krai in Russia (north-eastern Black Sea coast). The comparison revealed that the *Salmo* populations from the Black Sea coast of Anatolia are different from *Salmo labrax* from the Crimea Peninsula and the Caucasian coast in Russia. *Salmo trutta abanticus* was evaluated at species level; the two other populations were asserted morphologically distinct, diagnosable, and described as new species: *Salmo coruhensis* Turan, Kottelat & Engin, 2010 and *Salmo rizeensis* Turan, Kottelat & Engin, 2010. They also suggested that both species in several streams are present in sympatry, although rarely in syntopy. An opinion was proposed by Turan et al. (2010) that the trouts of the Black Sea basin in northeastern Turkey may represent distinct species as upstream resident trouts of different river drainages are genetically closer to each other than to migratory trouts in respective drainages. Tortonese (1955) reported both *Salmo trutta labrax* and *Salmo trutta macrostigma* from the Çoruh drainage. Turan et al. (2010) concluded that these apparently correspond to *Salmo coruhensis* and *Salmo rizeensis* respectively. The two species were also recorded from other streams and rivers draining to south-eastern Black Sea coast (see Turan et al. 2010: 336) (Fig. 1). *Salmo labrax* is distributed in the northern drainages of the Black Sea coast (undoubtly from the northwest Caucasia in Russia to the Danube River) but the exact border between the ranges of *Salmo labrax* and *Salmo coruhensis* is still not known and the trout from rivers in Georgia still needs a taxonomic study. As to *Salmo coruhensis*, it is distributed in the drainages of south-eastern Black Sea coast from the Çoruh River in the north to the Kızılırmak River.

Distribution of *Salmo macrostigma* is restricted to Algeria (Kottelat 1997; Delling and Doadrio 2005). The other peri-Mediterranean populations referred to as *Salmo macrostigma* belong to several species (e.g. *Salmo cettii* in Italy and *Salmo farioides* in the eastern Adriatic drainages) (Kottelat 1997). In Turkey, besides *Salmo rizeensis*, some populations earlier misidentified as *Salmo trutta macrostigma* were described as *Salmo tigridis* Turan, Kottelat & Bektaş, 2011 (Tigris-Euphrates drainage, Persian Gulf basin), *Salmo labecula* Turan, Kottelat & Engin, 2012 (Ecemiş Stream in the lower Seyhan River, east Mediterranean basin), Kartoz and Zindan streams (tributaries of Köprü Stream, a Mediterranean coastal drainage), *Salmo opimus* Turan, Kottelat & Engin, 2012 (Tekir, Fırnız and Göçüksu streams in lower reaches of the Ceyhan River) and Alara Stream (a Mediterranean coastal drainage), *Salmo chilo* Turan, Kottelat & Engin, 2012 (Akdere Stream in the upper Ceyhan River), *Salmo okumusi* Turan, Kottelat & Engin, 2014 (western Euphrates drainage), and *Salmo euphrataeus* Turan, Kottelat & Engin, 2014 (northeastern Euphrates drainage).

The taxonomic status of *Salmo* populations found in the southern Marmara Sea coast and the Trace Region will be discuss in forthcoming papers.

The present paper reports our data on the identity of the resident trout inhabiting Alakır, a small stream draining to the Mediterranean Sea. We conclude that it is an unnamed species. Here, it is described as a new species *Salmo kottelati*.

## Material and methods

Fish were caught with pulsed DC electro fishing equipment. Material is deposited in: FFR, Zoology Museum of the Faculty of Fisheries, Recep Tayyip Erdoğan University, Rize, and CMK, the collection of Maurice Kottelat, Cornol. Measurements and counts were all obtained on wild caught specimens, well preserved, in a straight position. Specimens not fixed straight or damaged were excluded. Most samples include both sexes, juveniles and mature specimens. Most *Salmo* populations are small, geographically restricted and under great threat because of overfishing and habitat destruction, and it is not advisable to collect and preserve large series of individuals. Colour pattern and variation in shape were observed in the field on additional individuals which were not preserved.

Measurements were taken with digital calipers (0.1 mm accuracy). Counts and measurements follow Hubbs and Lagler (1947), except as follows. Head depth 1: through eye; head depth 2: head depth at occiput; body depth1: body depth at dorsal-fin origin; body depth 2: body depth at anal-fin origin; body width: body width at level of anal-fin origin; adipose-fin height: measured at middle of fin base; length of adipose-fin base: measured from origin to insertion; length of caudal peduncle: measured from anal fin inseriton to middle of caudal-fin base; distance between adipose fin and caudal fin: measured from adipose-fin insertion to middle of caudal-fin base; length of maxilla: from anterior end to posterior end of upper margin of exposed part; length of mouth gape: maximum depth of combined maxilla and supramaxilla; width of mouth gape: measured between corners of mouth gape; length of mouth gape: distance from tip of snout to corner of mouth gape. Lateral-line scales were counted until the posterior extremity of the hypural complex. Scale rows between the adipose fin and the lateral line were counted at the adipose fin insertion. The last two branched dorsal and anal fin rays articulating on a single pterygiophore were counted as 1½. Vertebrae counts were obtained from radiographs and were counted separately as abdominal and caudal vertebrae. Abdominal vertebrae were counted from the first vertebra. The first caudal vertebra is that with its haemal spine fully developed. The count of caudal vertebrae includes the hypural complex. Sex was determined by examination of the gonads. In the description of colour pattern, we use *bands* to refer to the broad blackish vertical marks on the body, typically positioned behind the gill opening, below the dorsal fin, above the anal fin, and on the caudal peduncle. In some species these bands are known in well preserved specimens only, or in stressed individuals only, and in other species they are visible in situ in undisturbed individuals. A spot is called ocellated when surrounded by a white or very pale ring.

The morphometric and meristic data for *Salmo coruhensis*, *Salmo rizeensis*, *Salmo abanticus*, *Salmo caspius*, *Salmo tigridis*, *Salmo platycephalus*, *Salmo labecula*, *Salmo opimus*, *Salmo chilo*, *Salmo okumusi*, and *Salmo euphrataeus* are from Turan et al. (2010, 2011, 2012, 2014).

## Results

### Salmo kottelati sp. n.

Taxon classificationAnimaliaSalmoniformesSalmonidae

http://zoobank.org/A1CED992-9A64-4369-B88C-918DFB12BBDC

Fig. 1

#### Holotype.

FFR 03180, 205 mm SL, male; Turkey: Antalya Province: Altınyaka village; Alakır Stream (40°35.32'N, 40°51.50'E); D. Turan, E. Doğan and C. Kaya. 21 September 2014.

#### Paratypes.

FFR 03181, 21, 98–210 mm SL; same data as holotype. FFR 03182, 16, 98–176 mm SL; CMK 22405, 4, 97–143 mm SL; Turkey: Antalya Province: Altınyaka village; Alakır Stream (40°35.32'N, 40°51.50'E); M. Kanyılmaz, 15 September 2008.

#### Diagnosis.

*Salmo kottelati* is distinguished from all the described species of *Salmo* in Turkey by the combination of the following characters: 7–9 parr marks along lateral line distinct in males up to at least 176 mm SL and in females up to at least 208 mm SL; absence of four dark bands on flank in males and females; black spots on body numerous, ocellated, scattered on back, middle and upper part of flank (sometimes lower part of flank) in males larger than about 160 mm SL, and females between about 160−190 mm SL; in males and females smaller than about 160 mm SL, black spots few, present only on upper part of flank; few to numerous ocellated red spots on back and half of upper and lower flank; number of both black or red spots commonly increasing with size and age in males while number of both black and red spots decreasing with size and age in females; head long (29–33% SL in males, 26–32 in females); mouth large (length of mouth gape 13–19% SL in males, 12–15 in females), slightly subterminal; maxilla long (10–13% SL in males, 8–12 in females), reaching beyond eye in males longer than about 120 mm SL and in females longer than about 170 mm SL; 105–113 lateral-line scales (until posterior hypural margin); 24–29 scale rows between lateral line and dorsal-fin origin; 17–19 scale rows between lateral line and anal-fin origin; 13–15 scale rows between lateral line and adipose-fin insertion; gill rakers 18–20 on outer side of first gill arch.

#### Description.

General appearance shown in Fig. 1, morphometric and meristic data given in Tables 1 and 2. Dorsal profile of body behind head markedly convex, ventral profile less arched than dorsal profile in both sexes. Body moderately deep. Head long, 1.2–1.4 times body depth at dorsal-fin origin in males, 1.0–1.2 in females, slightly flattened dorso-ventrally in males longer than about 190 mm SL, not flattened in males smaller than about 180 mm SL, and in females. Head shape sexually dimorphic: upper profile slightly convex in interorbital area and convex on snout slightly behind level of nostrils in males, straight in interorbital area and markedly convex on snout in females. Mouth large, terminal or slightly subterminal in males larger than about 190 mm SL, subterminal in males smaller than about 180 mm SL and in females, conspicuously subterminal in juveniles. Upper jaw equal or slightly longer than lower jaw in males larger than about 190 mm SL, longer in females and juvenile, and in males smaller than about 180 mm SL. Tip of lower jaw slightly curved upward in males larger than about 190 mm SL and but in contrast, not curved in females of all sizes. Maxilla long, reaching beyond eye in males larger than about 120 mm SL and in females longer than about 170 mm SL, upper edge convex posteriorly in males, straight or slightly convex in females. Snout pointed in males and slightly pointed in females.

**Table 1.** Morphometric characters of *Salmo kottelati*. Number in parentheses: mean.

	*Salmo kottelati*				
Basin	Mediterranean Sea				
Drainage	Alakır Stream				
Province	Antalya				holotype
Sex and number of specimens	Males, n=20		Females, n=20		Male
	Range	SD	Range	SD	
Standard length (mm)	122–210		98–208		205
**In percentage of standard length**					
Head length	29.2–32.7 (30.9)	0.90	26.2–31.5 (28.7)	1.46	31.8
Predorsal length	48.9–52.4 (50.1)	1.13	46.7–50.7 (49.4)	1.34	52.3
Prepelvic length	53.9–60.3 (55.7)	1.43	50.1–56.7 (54.7)	1.31	57.6
Preanal length	72.8–83.1 (75.5)	2.07	73.2–82.8 (76.0)	1.93	75.6
Body depth at dorsal-fin origin	21.6–26.7 (24.4)	1.32	23.0–27.0 (25.4)	1.13	24.0
Body depth at anal-fin origin	15.8–20.6 (18.0)	1.16	17.6–20.5 (18.8)	0.82	18.9
Depth of caudal peduncle	10.1–13.3 (11.3)	0.80	10.5–12.9 (11.5)	0.76	12.0
Length of caudal peduncle	15.3–19.3 (16.7)	0.99	14.8–18.4 (17.0)	0.93	16.2
Distance between adipose and caudal fins	14.1–17.0 (15.0)	0.78	13.8–16.2 (14.9)	0.64	14.2
Body width at anal-fin origin	5.7–11.8 (8.7)	1.86	6.9–11.6 (9.2)	1.83	10.3
Length of dorsal-fin base	13.4–19.6 (14.7)	1.44	11.4–15.3 (14.0)	0.99	15.2
Depth of dorsal fin	15.0–22.4 (19.1)	2.06	17.3–22.4 (19.6)	1.56	19.6
Length of pectoral fin	19.8–24.5 (22.2)	1.35	19.8–25.2 (22.5)	1.55	20.2
Length of adipose-fin base	2.7–4.6 (3.8)	0.57	2.3–4.4 (3.6)	0.50	3.4
Depth of adipose fin	7.4–10.5 (8.7)	0.73	5.6–9.4 (8.0)	0.82	9.1
Length of pelvic fin	15.2–19.4 (16.9)	1.12	15.9–18.9 (17.2)	0.94	16.2
Depth of anal fin	17.3–20.6 (19.1)	0.89	17.7–21.3 (19.1)	1.03	19.1
Length of anal fin-base	7.7–11.7 (10.0)	0.86	8.9–14.1 (10.5)	1.36	9.8
Length of upper caudal-fin lobe	17.7–22.4 (19.8)	1.28	16.8–22.0 (19.3)	1.50	18.1
Length of median caudal-fin rays	13.3–16.9 (15.2)	0.89	13.6–17.6 (14.6)	0.87	14.6
Length of lower caudal-fin lobe	17.1–22.2 (19.8)	1.56	17.5–21.9 (20.1)	1.30	18.5
Snout length	6.7–9.9 (8.1)	0.83	6.3–8.9 (7.6)	0.77	9.9
Distance between nasal openings	4.5–6.3 (5.0)	0.49	4.4–6.1 (4.7)	0.65	5.9
Eye diameter	6.5–9.1 (7.6)	0.81	5.9–8.2 (7.1)	0.73	6.5
Interorbital width	6.5–9.6 (8.0)	0.93	6.3–9.0 (7.5)	0.74	6.8
Head depth through eye	13.7–16.8 (14.8)	0.73	11.9–14.9 (13.6)	0.90	16.8
Head depth at nape	18.4–20.3 (19.2)	0.54	16.3–19.7 (18.4)	0.92	18.6
Length of maxilla	10.1–13.3 (11.8)	0.87	8.2–11.7 (9.8)	0.91	12.6
Maximum height of maxilla	3.0–3.9 (3.4)	0.30	2.6–4.0 (3.3)	0.46	3.4
Width of mouth gape	10.6–12.9 (11.6)	0.69	9.3–12.6 (10.4)	0.89	12.6
Length of mouth gape	13.2–18.9 (15.6)	1.35	11.8–14.5 (13.1)	0.76	18.9

**Table 2.** Frequency of occurrence of meristic values in five *Salmo* species distributed in Mediterranean drainages of southern Anatolia.

Number of lateral-line scales to hypural margin																		
	n	105	106	107	108	109	110	111	112	113	114	115	116	117	118	119	120	mean
*Salmo kottelati*	36	2	4	2	3	4	10	1	8	2								109.4
*Salmo platycephalus*	20						1	2	5	7	2	1	2					112.9
*Salmo opimus*	27								2	4	8	4	3	2	2	1	1	114.5
*Salmo labecula*	17					2	4	2	3	5		1						111.6
*Salmo chilo*	19				2	3	2	2	5	3	2							111.1
**Transverse scale rows**																		
**Above lateral line**	**n**	**23**	**24**	**25**	**26**	**27**	**28**	**29**	**mean**									
*Salmo kottelati*	36		6	10	8	7	3	2	25.9									
*Salmo platycephalus*	20	6	6	8					24.1									
*Salmo opimus*	27		6	12	9				25									
*Salmo labecula*	17	11	2	5					23.5									
*Salmo chilo*	19		2	9	8				25.3									
**Below lateral line**	**n**	**15**	**16**	**17**	**18**	**19**	**mean**											
*Salmo kottelati*	36			9	7	20	18.3											
*Salmo platycephalus*	20			14	6		17.3											
*Salmo opimus*	27		10	13	14		17.3											
*Salmo labecula*	17		10	7			16.4											
*Salmo chilo*	19	4	6	9			16.3											
**Scale rows between adipose-fin insertion and lateral-line**																		
	**n**	**13**	**14**	**15**	**mean**													
*Salmo kottelati*	36	8	19	9	14													
*Salmo platycephalus*	20	4	8	8	14.2													
*Salmo opimus*	27	10	13	4	13.8													
*Salmo labecula*	17		8	9	14.5													
*Salmo chilo*	19	5	14		13.7													
**Gill rakers**																		
	**18**	**19**	**20**	**21**	**22**	**23**	**24**	**25**	**mean**									
*Salmo kottelati*	5	20	11						19.2									
*Salmo platycephalus*						7	9	4	23.9									
*Salmo opimus*	4	8	12	3					19.5									
*Salmo labecula*				2	10	5			22.2									
*Salmo chilo*		5	14						19.7									

Lateral line with 105–113 scales; 24–29 scale rows between lateral line and dorsal-fin origin; 17–19 scale rows between lateral line and anal-fin origin; 13–15 scale rows between lateral line and adipose-fin insertion (Table 2). Dorsal fin with 3–4 simple and 9½–10½ branched rays, outer margin straight or slightly concave. Adipose fin small in males smaller than about 170 mm SL with upper edge straight or slightly convex anteriorly and convex posteriorly or large, almost reaching caudal-fin base in males larger than about 190−200 mm SL, upper margin markedly convex. In females, adipose fin small to medium, upper edge markedly convex both anteriorly and posteriorly. Pectoral fin long, with 1 simple, 10–13 branched rays, outer margin slightly concave. Pelvic fin with 1 simple and 7–9 branched rays, outer margin slightly convex. Anal fin with 3 simple and 6½–8½ branched rays, outer margin straight or slightly convex anteriorly, concave posteriorly. Caudal fin long and emarginate in small to large size specimens, lobes slightly pointed. Gill rakers 6–7 + 12–13 = 18–20 on outer side of first gill arch. Total vertebrae 55(2), 56(12), 57(5), and 58(1); predorsal vertebrae 13–15 with mode of 14; number of abdominal vertebrae 33–35 with mode of 34, and that of caudal vertebrae 21–23 with mode of 22. Abdominal region longer than caudal region, rarely regions equal, and difference between abdominal and caudal counts 11–14; most common vertebral formula 34+22.

**Sexual dimorphism.** Males of *Salmo kottelati* having longer head and maxilla and greater mouth gape than females.

#### Colouration.

General body colour greenish to silvery in life. Body dark brown on back and upper part of flank, brownish to yellowish on lower part of flank and belly yellowish in preserved specimens (Fig. 1). A large black spot (larger than pupil but smaller than eye) and one to five small black spots (smaller than pupil) behind eye (on cheek and preopercle), and 4–15 on opercle (smaller than pupil). Three to 21 black spots on top of head, smaller than pupil. Black spots on body, numerous, ocellated, medium to large (equal to or smaller than pupil), scattered on back (sometimes present in predorsal area), middle and upper part of flank (sometimes lower part of flank) in males larger than about 160 mm SL. Black spots few, present only on upper part of flank in both sexes smaller than about 160 mm SL, and in females over about 190 mm SL. Red spots few to numerous, ocellated, scattered on median, and half of lower and upper part of flank. Number of both black or red spots increasing with size and age in males except for one male (Fig. 1B) but, in contrast, number of both black and red spots decreasing with size and age in females. Dorsal fin greyish, with three or four rows of red spots posteriorly on lower part, 6–7 rows of black spots on middle, lower and upper part, boldly marked. Caudal fin dark grey, outer margin blackish. Pectoral, pelvic and anal fins yellowish. Leading edge of anal fin white in specimens larger than about 170 mm SL. Adipose fin greyish. Seven to nine parr marks distinct in males up to at least 176 mm SL and in females up to at least 208 mm SL, broad and large (Fig. 1), slightly rounded.

#### Distribution and habitat.

**Figure 2.**
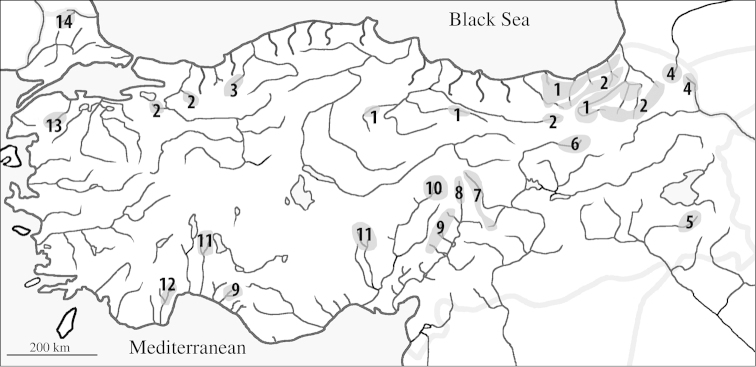
Distribution of *Salmo* species in Anatolia: *Salmo coruhensis* (**1**) *Salmo rizeensis* (**2**) *Salmo abanticus* (**3**) *Salmo caspius* (**4**) *Salmo tigridis* (**5**) *Salmo euphrataeus* (**6**) *Salmo okumusi* (**7**) *Salmo chilo* (**8**) *Salmo opimus* (**9**) *Salmo platycephalus* (**10**) *Salmo labecula* (**11**) *Salmo kottelati* (**12**) Salmo cf. coruhensis (**13**) Salmo cf. rizeensis (**14**).

*Salmo kottelati* is only known from Alakır Stream in which located about 96 km southwest of the city of Antalya, a drainage of Mediterranean Sea in southern Anatolia (Fig. 2). It inhabits in cold and clear water and moderate current, with gravel and pebble substrate.

#### Etymology.

The species is named for Maurice Kottelat, who contributed to the knowledge of the fish fauna of Europe and Asia.

## Discussion and comparisons

*Salmo kottelati* is immediately distinguished from all other species of *Salmo* in Turkey and *Salmo labrax* (from the northern Black Sea basin) by having fewer parr marks on the flank (7–9, vs. 10–13). It is further distinguished from *Salmo platycephalus*, *Salmo opimus*, *Salmo chilo*, and *Salmo labecula* by the absence of four dark bands on the flank in males and females (vs. presence).

Besides the above differences, *Salmo kottelati* is distinguished from *Salmo platycephalus* by the presence of red spots on the flank in individuals of all sizes (vs. absence in specimens larger than about 70 mm SL) and the presence of black spots in individuals of all sizes (vs. absence in specimens larger than about 170 mm SL). The new species has fewer gill rakers on the outer side of the first gill arch (18–20, vs. 23–25), a longer head in males (29–33% SL vs. 27–29), a deeper head (depth through eye 14–17% SL in males, 12–15 in females, vs. 12–13 in males, 11–12 in females), and a longer maxilla (10–13% SL in males, 8–12 in females, vs. 8–10 in males, 7–8 in females).

Besides the differences listed above, *Salmo kottelati* differs from *Salmo opimus* in having fewer lateral-line scales (105–113, vs. 112–120) and by the number and location of the black and red spots on the body in males and in females larger than about 160 mm SL. In *Salmo kottelati*, the black spots are numerous in males larger than about 160 mm SL and in females between about 160–190 mm SL, present on median, and half of upper and lower of part flank, and the number of both red and black spots increases with increasing size and age in males. In *Salmo opimus*, black spots are few (less than 50), present on back and upper part of flank, and their number does not increase with increasing size and age in males. There are also other differences between males of *Salmo kottelati* and *Salmo opimus*: males of *Salmo kottelati* have a deeper head (head depth through eye 14–17% SL, vs. 12–13), a longer maxilla (10–13% SL, vs. 9–10), and a greater mouth gape (length of gape 13–19% SL, mean 16, vs. 11–14, mean 13).

In addition to the differences mentioned above, *Salmo kottelati* is distinguished from *Salmo chilo* by the following characters: a slightly convex dorsal profile of head (vs. strongly convex), a pointed snout (vs. blunt), a slightly subterminal mouth (vs. conspicuously subterminal), non-flesh maxilla and lower lip (vs. flesh), length of the maxilla markedly sexually dimorphic (the length in males markedly longer than that in females, vs. not sexually dimorphic); black spots on the body roundish (vs. irregularly shaped). The new species also differs from *Salmo chilo* by having more numerous scale rows between the lateral line and the anal-fin origin (17–19, vs. 15–17). When compared to males of *Salmo chilo*, males of *Salmo kottelati* have a deeper head (depth through eye 14–17% SL, vs. 12–13), and a longer maxilla (length 10–13% SL, mean 12, vs. 9–10, mean 9).

Besides the differences mentioned above, *Salmo kottelati* differs from *Salmo labecula* by the presence of red spots on the flank (vs. absence in specimens larger than about 70 mm SL) and the black spots on body roundish (vs. irregularly shaped). *Salmo kottelati* has more numerous scale rows between the lateral line and the anal-fin origin (17–19, vs. 16–17) and fewer gill rakers (18–20, vs. 21–23). It also differs from *Salmo labecula* by having a greater predorsal distance (49–52% SL, mean 50 in males, 47–51, mean 49 in females, vs. 45–48% SL, mean 46 in males, 44–47, mean 45 in females). Moreover, *Salmo kottelati* has a longer head in males (29–33% SL, mean 31, vs. 27–29, mean 28) and a deeper head in males (depth through eye 14–17% SL, mean 15, vs. 11–14, mean 13).

*Salmo kottelati* is further distinguished from *Salmo tigridis* by the number and locations of black spots on the body in specimens larger than about 160 mm SL. In *Salmo kottelati*, black spots are numerous in males larger than about 160 mm SL and in females between about 160–190 mm SL, present on median and half of upper and lower part of flank, and the number of both red and black spots increases with increasing size and age in males. In *Salmo tigridis*, black spots are few (less than 50), present on back and the upper part of flank and their number does not increase with increasing size and age. The new species also differs from *Salmo tigridis* by having fewer scale rows between the lateral line and the dorsal-fin origin (24–29, vs. 32–35), fewer scale rows between the lateral line and the anal-fin origin (17–19, vs. 22–26), and fewer scale rows between the adipose fin insertion and the lateral line (13–15, vs. 19–20). Besides the above listed differences, *Salmo kottelati* males are distinguished from *Salmo tigridis* males by a longer (29–33% SL, mean 31, vs. 25–28, mean 27) and deeper head (depth at nape 18–20% SL, mean 19, vs. 17–18, mean 17), a longer maxilla (10–13% SL, mean 12, vs. 8–9, mean 9), and a wider mouth gape (11–13% SL, mean 12, vs. 9–10, mean 10).

*Salmo kottelati* is further distinguished from *Salmo abanticus* by the presence of red spots on the body in specimens larger than about 210 mm SL (vs. absent), the shape of the black spots on the flank (round, vs. polygonal), the shape of the ring around black spots (circular, vs. polygonal), and the size of the black spots (about equal to the pupil, vs. markedly larger than the pupil). It has fewer lateral line scales than *Salmo abanticus* (105–113, vs. 113–121). Besides the differences mentioned above, it also differs from *Salmo abanticus* in having a longer head (29–33% SL, mean 31 in males, 26–32, mean 29 in females, vs. 26–29, mean 27 in males, 24–26, mean 26 in females). Males of *Salmo kottelati* differ from males of *Salmo abanticus* in having a greater predorsal distance (49–52% SL, vs. 47–48), a longer maxilla (10–13% SL, mean 12, vs. 9–10, mean 10), a greater eye diameter (7–9% SL, mean 8, vs. 6–7, mean 6), and a deeper head at nape (18–20% SL, mean 19, vs. 17–19, mean 17).

*Salmo kottelati* is further distinguished from *Salmo coruhensis* by fewer scale rows between the anal-fin origin and the lateral line (17–19, vs. 19–23), fewer scale rows between the adipose fin insertion and the lateral line (13–15, vs. 15–17), a longer (29–33% SL, mean 30.9, vs. 26–28, mean 27.3) and deeper head in males (depth through eye 14–17% SL, mean 15, vs. 12–14, mean 13).

*Salmo kottelati* is further distinguished from *Salmo rizeensis* by the number and location of black spots on the body. In *Salmo kottelati*, black spots are numerous (60 and more) in males larger than about 160 mm SL and in females between about 160–190 mm SL, present on median, and half of upper and lower of part flank, and the number of both red and black spots increases with increasing size and age in males. In *Salmo rizeensis*, black spots are few (less than 40), present on back and upper part of flank, and their number does not increase with increasing size and age. The new species also differs from *Salmo rizeensis* by the general body colour (greenish to silvery in life, vs. brownish), the adipose fin almost reaching the base of the caudal fin in males larger than 200 mm SL (vs. not reaching in all-sized specimens), and fewer lateral-line scales (105–113, vs. 114–120).

*Salmo kottelati* is further distinguished from *Salmo caspius* from the upper Kura drainage by having fewer lateral line scales (105–113, vs. 112–119), fewer scale rows between the lateral line and the anal-fin origin (17–19, vs. 19–22) and fewer scale rows between the insertion of the adipose fin and the lateral line (13–15, vs. 15–17). In *Salmo kottelati*, the general body colour is greenish to silvery in life (vs. brownish). Morover, males of *Salmo kottelati* have a deeper head than that of *Salmo caspius* (head depth at nape 18–20% SL, vs. 17–18).

*Salmo kottelati* is further distinguished from *Salmo okumusi* by the absence of four dark bands on the flank in males and females (vs. very faintly marked or indistinct in small specimens but distinct in specimens larger than about 230 mm SL); the parr marks vertically oblong (vs. vertically elongate); the black spots circular (vs. irregularly shaped), bigger black spots on dorsal fin (slightly smaller than pupil, vs. smaller than half pupil). *Salmo kottelati* has a longer head than *Salmo okumusi* (29–33% SL in males, 26–32 in females, vs. 26–27 in males, 25–26 in females). Males of *Salmo kottelati* differs from males of *Salmo okumusi* by having a longer maxilla (10–13% SL, vs. 9–10) and a deeper head (at nape 18–20% SL, vs. 16–17).

*Salmo kottelati* is further distinguished from *Salmo euphrataeus* by the general body colour and the number and position of the black spots on the body: in *Salmo kottelati*, black spots are commonly numerous in males and increasing the number with size (vs. few and not increasing the number) and located on median, and half of upper and lower parts of the flank (vs. restricted to the upper part of the flank, mostly in its anterior area). The new species also differs from *Salmo euphrataeus* in having fewer lateral-line scales (105–113, vs. 112–120), fewer scale rows between the dorsal-fin origin and the lateral line (24–29, mean 26, vs. 28–31, mean 29), and fewer scale rows between the anal-fin origin and the lateral line (17–19, vs. 19–23).

*Salmo kottelati* differs from resident *Salmo labrax* by a large black spot behind the head (larger than the pupil but smaller than the eye, vs. equal or smaller than the pupil), black spots on the body smaller than the pupil (vs. larger), more numerous red spots on the body (red spots few to numerous, scattered on median, and half of lower and upper part of the flank, vs. few, one or two irregularly rows of spots, scattered along the lateral line or, sometimes, below it). *Salmo kottelati* has fewer lateral line scales (105–113, vs. 112–121), fewer scale rows between the dorsal-fin origin and the lateral line (24–29, vs. 28–32), fewer scale rows between the lateral line and the anal-fin origin (17–19, vs. 19–23), fewer scale rows between the adipose fin insertion and the lateral-line (13–15, vs. 15–16), and more numerous gill rakers (18–20, vs. 16–18). Males of *Salmo kottelati* are further distinguished from males of *Salmo labrax* by having a longer head (29–33% SL, vs. 25–28), a greater predorsal length in males (49–52% SL, vs. 46–47), a deeper head (depth through eye 14–17% SL, vs. 11–13), a longer maxilla (10–13% SL, vs. 9–10), a wider mouth gape (11–13% SL, vs. 8–10), and a longer mouth gape (13–19% SL, mean 16, vs. 12–14, mean 13).

*Salmo kottelati* can be further distinguished from Salmo cf. coruhensis from the Gönen Stream (southern Marmara Sea) by fewer lateral-line scales (105–113, vs. 115–121), fewer scale rows between the lateral line and the anal-fin origin (17–19, vs. 20–23), fewer scales between the adipose fin insertion and the lateral line (13–15, vs. 15–17), a larger adipose fin (depth 7–11% SL, vs. 4–7), and a deeper caudal peduncle (depth 10–13% SL, vs. 8–10). Males of *Salmo kottelati* differs from males of Salmo cf. coruhensis by a longer head (29–33% SL, vs. 24–28), a greater predorsal distance (49–52% SL, 45–47), a longer mouth gape (13–19% SL, mean 16, vs. 11–14, mean 12), a narrower mouth gape (width 11–13% SL, vs. 8–10), a longer maxilla (10–13% SL, vs. 8–10), and a deeper head (depth through eye 14–17% SL, vs. 11–14).

*Salmo kottelati* is further distinguished from Salmo cf. rizeensis from the Rezova Stream (Trace Region) by fewer lateral-line scales (105–113, vs. 114–121), fewer scale rows between the dorsal-fin origin and the lateral line (24–29, vs. 29–34), fewer scale rows between the lateral line and anal-fin origin (17–19, vs. 20–23), fewer scales between the adipose fin insertion and the lateral line (13–15, vs. 16–17), a larger adipose fin (depth 7–11% SL, vs. 5–7), and a deeper caudal peduncle (depth 10–13% SL, vs. 9–10). Males of *Salmo kottelati* differs from males of Salmo cf. rizeensis by a longer (29–33% SL, vs. 26–29) and deeper head (depth through eye 14–17% SL, vs. 12–14).

### Comparison material

*Salmo platycephalus*: 34, 75–550 mm SL; Turkey: Kayseri Prov.: Pınarbaşı Stream in Pınarbaşı district, Seyhan River drainage.

*Salmo chilo*: 33, 65–235 mm SL; Turkey: Sıvas Prov.: Akdere Stream at Gürün county, Ceyhan River drainage.

*Salmo labecula*: 19, 85–400 mm SL, male; Turkey: Niğde Prov.: Ecemiş Stream at Çamardı county, Seyhan River drainage. 10, 140–241 mm SL; Turkey: Isparta prov.: Kartoz Köprüçay.

*Salmo opimus*: 13, 118–180 mm SL; Turkey: Antalya Prov.: Alara Stream at Gündoğmuş. 25, 115, 147–186 mm SL; mm SL; Turkey: Kahramanmaraş prov.: Göçüksu Stream at Kömürköy, Ceyhan River drainage. 4, 175–210 mm SL; Turkey: Kahramanmaraş Prov.: Tekir Stream at Tekir, Ceyhan River drainage. 9, 90–300 mm SL; Turkey: Kahramanmaraş Prov.: Fırnız Stream at Fırnız, Ceyhan River drainage.

*Salmo tigridis*: 13, 136–227 mm SL; Turkey: Van Prov.: Çatak Stream, Tigris River drainage. 7, 15–18 mm SL, Turkey: Van Prov.: Müküs Stream, Tigris River drainage.

*Salmo rizeensis*: 16, 88–237 mm SL; Turkey: Erzurum Prov.: Ovit(2) [Kan] Stream at Ovit mountain, Çoruh River drainage. 7, 88–237 mm SL; Turkey: Artvin Prov.: Dörtkilise Stream at Tekkale Village, Çoruh River drainage. 12, 75–167 mm SL; Turkey: Artvin Prov.: Çifteköprü Stream at Cankurtaran mountain, Çoruh River drainage. 11, 113–221 mm SL; Turkey: Erzurum Prov.: Yağlı Stream at Yağlı village, Çoruh River drainage. 16, 145–224 mm SL; Turkey: Giresun Prov.: Akbulak stream at Akbulak village, Yeşilırmak River drainage. 10, 122–221 mm SL; Turkey: Kütahya Prov.: Sefaköy Stream at Domaniç, Sakarya River drainage. 10, 111–119 mm SL; Turkey: Kütahya Prov.: Çatalalıç Stream at Domaniç, Sakarya River drainage. 13, 111–220 mm SL; Turkey: Rize Prov.: Çağlayan Stream at Gürcüdüzu figau. 18, 95–226 mm SL; Turkey: Rize Prov.: Şehitlik Stream at Şehitlik village. 12, 90–118 mm SL; Turkey: Rize Prov.: Çayeli Stream at Kaptanpaşa village. 10, 90–238 mm SL; Turkey: Rize Prov.: Ovit Stream at Ovit mountain, İyidere drainage. 14, 120–200 mm SL; Turkey: Rize Prov.: Fırtına Stream on Elevit Plateau. 10, 114–245 mm SL; Turkey: Trabzon Prov.: Değirmen Stream at Çoşandere village. 12, 112–230 mm SL; Turkey: Trabzon Prov.: Solaklı Stream at Demirkapı village.

*Salmo coruhensis*: 13, 90–380 mm SL; Turkey: Erzurum Prov.: Uzundere district; Pehlivanlı Stream at Pehlivanlı village [tributary of Tortum], Çoruh River drainage. 13, 115–330 mm SL; Turkey: Artvin Prov.: Dörtkilise Stream at Tekkale village, Çoruh River drainage. 5, 130–229 mm SL; Turkey: Artvin Prov.: Barhal Stream at Sarıgöl village, Çoruh River drainage. 16, 190–465 mm SL; Turkey: Bayburt Prov.: Ölçer Stream at Ölçer village, Çoruh River drainage. 17, 80–550 mm SL Turkey: Erzurun Prov.: Çayırbaşı (Kırık) Stream at Kırık village, Çoruh River drainage. 6, 160–290 mm SL; Turkey: Erzurum Prov.: Madenköprübaşı district; Büyük Stream at Büyükköy village, Çoruh River drainage. 17, 70–210 mm SL; Turkey: Gümüşhane Prov.: Harşut Stream at Yağmurdere village. 6, 95–117 mm SL; Turkey: Rize Prov.: Sarayköy Stream at Sarayköy village. 6, 100–250 mm SL; Turkey: Rize Prov.: İyidere Stream in İyidere district. 7, 150–450 mm SL; Türkey: Rize Prov.: Fırtına Stream at Çamlıhemşin. 5, 10–280 mm SL; Türkiye: Rize Prov.: Limanköy Stream at Limanköy village. 25, 90–520 mm SL; Türkiye: Rize Prov.: Fırtına Stream at Çat village. 11, 95–228 mm SL; Turkey: Rize Prov.: Kendirli Stream at Kalkandere District on road to Kendirli village, İyidere drainage. 13, 120–450 mm SL; Turkey: Rize Prov.: İyidere Stream (İkizder) at Güneyce. 6, 130–420 mm SL; Turkey: Rize Prov., Veliköy Stream at Veliköy village. 9, 160–450 mm SL; Turkey: Sıvas Prov.: Gemin country, Yeşilırmak River drainage on road of Sıvas.

*Salmo abanticus*: 20, 113–300 mm SL; Turkey: Bolu Prov.: outlet of Lake Abant.

*Salmo caspius*: 10, 126–222 mm. SL; Turkey: Ardahan Prov.: Çataldere Stream at Ardahan, Kura River drainage. 30, 110–250 mm. SL; Turkey: Ardahan Prov.: Tora Stream at Ardıcdere village, Kura River drainage. 8, 135–240 mm SL; Turkey: Ardahan Prov.: Aşıkzülal Stream at Aşıkzülal village, Kura River drainage.

*Salmo labrax*: 6, 107–147 mm SL; Ukraine: Ula-Uzen River. 6, 102–160 mm SL; Russia: Krasnodar Prov.: Khosta River.

*Salmo okumusi*: 11, 75– 213 mm SL; Turkey: Malatya Prov.: Sürgü Stream, Euphrates River drainage. 33, 68–28 mm SL; Turkey: Sıvas Prov.: Gökpınar Stream (tributary of Tohma Stream], Euphrates River drainage.

*Salmo euphrataeus*: 36, 80–226 mm SL; Turkey: Erzurum Prov.: Kuzgun Stream (tributary of Karasu Stream), Euphrates River drainage. 18, 88–230 mm SL; Turkey: Erzurum prov.: Şenyurt Stream (tributary of Karasu Stream), Euphrates River drainage. 10, 160–250 mm SL; Turkey: Erzurum Prov.: Ağırcık Stream at Ağırcık Village (tributary of Karasu Stream), Euphrates River drainage. 12, 95–300 mm SL; Turkey: Erzurum Prov.: Sırlı Stream at Sırlı Village (tributary of Karasu Stream), Euphrates River drainage.

Salmo cf. coruhensis: 28, 95–228 mm SL; Turkey: Çanakkale Province: Çelebi Stream, drainage of Gönen Stream. 12, 108–160 mm SL; Turkey: Çanakkale Province: Kilise Stream; drainage of Gönen River.

Salmo cf. rizeensis: 50, 90–220 mm SL; Turkey: Kırklareli Province: Rezova Stream.

## Supplementary Material

XML Treatment for Salmo kottelati
